# The efficacy and potential mechanism of the acupuncture treatment for type 2 diabetes mellitus: A systematic review and meta-analysis of data from animal models

**DOI:** 10.3389/fendo.2025.1627061

**Published:** 2025-10-06

**Authors:** Linsun Lin, Shu Li, Ziyi Guo, Peigang Fang, Yanchen Feng, Congcong Qi, Mingyang Wang, Lu Xiao, Min Chen, Tao Wang

**Affiliations:** ^1^ Faculty of Chinese Medicine and State Key Laboratory of Quality Research in Chinese Medicines, Macau University of Science and Technology, Macao, Macao SAR, China,; ^2^ Huizhou Health Sciences Polytechnic, Huizhou, China; ^3^ The Third Affiliated Hospital of Zhengzhou University, Zhengzhou, Henan, China; ^4^ National Engineering Laboratory for Internet Medical Systems and Applications, The First Affiliated Hospital of Zhengzhou University, Zhengzhou, Henan, China; ^5^ Department of Rehabilitation Medicine, The First Affiliated Hospital of Zhengzhou University, Zhengzhou, Henan, China; ^6^ School of Traditional Chinese Medicine, Henan University of Chinese Medicine, Zhengzhou, Henan, China; ^7^ Zunyi Medical University, Zhuhai, China; ^8^ Guangdong Medical University, Zhanjiang, China; ^9^ Encephalopathy Center, The Second Affiliation Hospital of Anhui University of Chinese Medicine, Hefei, Anhui, China

**Keywords:** acupuncture, diabetes mellitus, blood glucose, meta-analysis, animal

## Abstract

**Background:**

Type 2 diabetes mellitus (T2DM) is a prevalent metabolic disorder with limited treatment options. Manual acupuncture and electroacupuncture have been investigated in numerous animal studies for their potential to improve glycemic and lipid profiles, but no comprehensive synthesis exists. This review aims to evaluate the effects of manual acupuncture and electroacupuncture on blood glucose and lipid levels in animal models of T2DM, and to explore potential mechanisms.

**Methods:**

A systematic search was conducted in PubMed, Embase, Cochrane, Web of Science, and major Chinese databases from inception to December 2024. Only animal studies employing manual acupuncture or electroacupuncture for T2DM models were included. The methodological quality was assessed using a 10-item CAMARADES checklist. Meta-analyses were performed using STATA 17.0, and subgroup analyses explored the influence of modeling methods, intervention timing, and treatment duration.

**Results:**

A total of 14 studies with 274 animals with T2DM were included. The overall quality of the included reports was rated as moderate or higher. Meta-analysis showed that acupuncture significantly reduced blood glucose [Standardized Mean Difference(SMD)= -3.15, 95% Confidence Interval(CI) (-4.18, -2.12), I-squared (*I*²)= 85.1%, (*P*< 0.05)], body weight [SMD = -3.36, 95%CI (-4.77, -1.95), *I*²= 84.2%,(*P*<0.05)], triglycerides [SMD=-2.50, 95% CI (-3.00, -2.01), *I*²= 0.0%, (*P*< 0.05)], total cholesterol [SMD = -2.60, 95% CI (-3.55,-1.65), *I*²= 74.9%, (*P*< 0.05)], and low-density lipoprotein [SMD = -3.36, 95%CI (-5.42,-1.95), *I*²= 86.2%] (*P*< 0.05)], and no statistically significant difference was observed in high-density lipoprotein [SMD = 0.61, 95% CI (-0.98, 2.19), *I*² = 92.1%, (*P*> 0.05)] compared to the control group. These results suggest that acupuncture can effectively improve blood glucose and lipid levels in animal models of T2DM.

**Conclusion:**

While this study is limited by the number of included studies, the results indicate that acupuncture can effectively improve blood glucose and lipid levels in animal models of T2DM.

**Systematic Review Registration:**

https://www.crd.york.ac.uk/prospero/, identifier CRD42024520000.

## 1 Introduction

T2DM is an endocrine and metabolic disorder disease, which is defined as being characterized by chronic hyperglycemia resulting from pancreatic β-cell dysfunction, insulin resistance, or both (1). At present, the growing number of T2DM patients than expected, with approximately 9.36% (529 million) people of all ages suffering from T2DM in 2021 (2), it is predicted that will rise to10.9% (700 million) in 2045 (3). Diabetes not only has a high morbidity rate but also cause a series of chronic complications such as diabetic nephropathy, diabetic retinopathy and so on, which affect people’s health and lead to the primary cause of death even in diabetic patients (4). As the prevalence and treatment costs of T2DM continue to increase globally, diabetes imposes a significant economic burden, which has become one of the most serious challenges to public health worldwide (5, 6). To address this global public health issue, current treatment strategies primarily involve the commonly used drugs for the treatment of T2DM are metformin and insulin, and metformin is often used as a first-line pharmacological treatment, which have shown good clinical efficacy in controlling blood sugar and are often considered the best initial treatment for diabetes mellitus (7). Although these medications can effectively control blood glucose levels in a fast-acting manner, long-term use of them can lead to side effects such as hypoglycemia, nausea, and nerve damage (8). Therefore, there is an urgent need for research to seek more effective and alternative therapies for the prevention and treatment of T2DM, particularly with respect to both efficacy and safety, which will alleviate the burden on both families and society in the long run (9).

T2DM is a complex metabolic disorder influenced by various genetic and environmental factors, affecting multiple organs such as the heart, nerves, and blood vessels (1). The pathogenesis of T2DM is characterized by several mechanisms, including dysfunction of pancreatic β-cells, insulin resistance, and impaired insulin processing (10). Recent studies have explored acupuncture as a traditional therapeutic modality for its potential effects on diabetes management (11). Research indicates that acupuncture can improve the expression of upstream and downstream proteins and genes associated with the PI3K signaling pathway, thereby influencing critical physiological functions such as cell growth, survival, and proliferation. This regulatory effect may assist in controlling glucose metabolism levels, leading to beneficial outcomes for diabetic patients (12). Insulin resistance and β-cell dysfunction are key factors in the development of diabetes. Acupuncture not only directly activates the vagus nerve to ameliorate local immune inflammatory responses in target organs but also regulates the activity of immune cells to achieve immune balance. Additionally, acupuncture may engage neuroendocrine pathways to participate in inflammation and immune regulation, further contributing to its hypoglycemic effects (13). Moreover, dysbiosis of gut microbiota has been recognized as an important pathogenic mechanism in diabetes. Studies have demonstrated that acupuncture can modulate and improve glucose and lipid metabolism as well as the structure of gut microbiota in mice, effectively regulating blood sugar levels and enhancing insulin sensitivity (14, 15). A common therapeutic strategy for both Type 1 and Type 2 Diabetes Mellitus is to address the absolute or relative deficiency of insulin. Evidence suggests that acupuncture can enhance the morphology and function of pancreatic β-cells by influencing the expression levels of related genes and proteins, thereby strengthening β-cell functionality. Consequently, acupuncture presents itself as a promising non-pharmacological alternative therapy for individuals with diabetes (16). In summary, acupuncture demonstrates potential in diabetes management through various mechanisms that regulate metabolism and immune responses, offering hope for improved patient outcomes.

In China, acupuncture is a key component of Traditional Chinese Medicine (TCM) and has been used as an alternative therapy to treat diseases for a long time. Since 1979, it is recognized as a complementary therapy by the World Health Organization (WHO) (17). Several systematic reviews and meta-analyses have evaluated the effects of acupuncture in patients with diabetes mellitus, generally reporting small sample sizes, methodological limitations, and insufficient evidence to make strong clinical recommendations. Our study focuses on animal models to provide preclinical evidence, which complements these human studies and can guide the design of future large-scale, high-quality clinical trials. Virtually, acupuncture has been used to treat diabetes mellitus because of its benefits in various aspects such as effectiveness, economy, convenience, and low side effect (18). Some modern medical research has confirmed that acupuncture can decrease blood sugar and improve the symptoms of diabetes because of its effects on enhancing the insulin sensitive index, ameliorating insulin resistance, and regulating blood lipids (11). In human clinical trials investigating acupuncture for diabetes, ethical constraints—such as the difficulty in designing ethical and effective sham control groups, challenges in achieving adequate blinding (which raises issues with informed consent), and the tension between standardized protocols and individualized treatment—significantly limit the methodological rigor and scope of studies. Consequently, these constraints impede the acquisition of comprehensive and unambiguous evidence regarding acupuncture’s specific efficacy. Animal studies not only allow for the minimization of external factors to attain more precise results but also facilitate the exploration of the experiment’s underlying mechanisms (19). A systematic review of animal studies can bring more precise accurate decisions in medical care (20). Therefore, we aim to a systematic review and meta-analysis of the effectiveness of acupuncture for treating diabetes in animal models by regulating lipid metabolism. whether acupuncture therapy the effects by regulating lipid metabolism for T2DM on levels of blood glucose (BG), high density lipoprotein (HDL), low-density lipoprotein (LDL), triacylglycerol (TG), Cholesterol (TC) in T2DM animals. overall, acupuncture may provide innovative therapeutic strategies to improve the clinical management of T2DM.

## 2 Methods and materials

This systematic review and meta-analysis were reported according to the PRISMA guidelines (21)and Cochrane Collaboration standard (22).

### 2.1 Search strategy

We systematically searched several major English-language electronic databases, including PubMed, EMBASE, the Cochrane Library, and Web of Science, from their inception until December 2023 with the language restricted to English. We searched for titles, abstracts, and keywords that used the following terms:((electroacupuncture[Mesh]) OR(acupuncture[Mesh])) AND ((diabetes[Mesh]) OR (diabetes mellitus[Mesh]) OR (Alloxan Diabetes[Keywords]) OR (Diabetes Mellitus, Experimental[Keywords])) Also, reference lists of the selected articles in the original search results were reviewed to obtain more relevant studies.

### 2.2 Inclusion/exclusion criteria

Study inclusion was based on the following criteria: subjects (rodent model of T2DM), intervention (acupuncture as the primary mode of treatment, only manual acupuncture and electroacupuncture interventions were included; other forms of acupuncture such as fire needling, warm needling, or intradermal needling were excluded to ensure methodological homogeneity), and outcome (mainly focused on assessing individual diabetes efficacy data such as animal blood glucose levels as the primary measure of acupuncture efficacy). Moreover, bodyweight and food intake data extracted from animal studies will be considered as the secondary outcomes. Studies failing to meet these specified criteria or failing to grant access to the complete text were excluded. Additionally, clinical trials not pertinent to the subject matter were omitted. Subsequently, following a comprehensive assessment of the complete text, studies diverging from the predetermined criteria concerning methodologies and reported findings were additionally eliminated.

### 2.3 Data extraction

Data extraction was carried out independently by two researchers, namely LLS and GZY, reviewed titles, authors, and publication dates to identify and exclude duplicate literature. They applied exclusion criteria to eliminate irrelevant studies, following this, they meticulously examined the full text to ascertain the final selection of included literature based on predetermined inclusion criteria. Finally, they proceeded to extract data from the selected literature. Extraction of data included the following variables: year of publication, first author name, type of diabetes model, specific disease or condition, sample size, type of acupuncture, and nature of specimen investigated. In addition to these parameters, average blood glucose levels within each group (e.g., disease group, intervention group) were extracted, along with corresponding measures of variability. Sham acupuncture refers to needle insertion at non-acupoints or superficial insertion without stimulation, designed to mimic the procedure without eliciting therapeutic effects. In our included studies, no sham acupuncture was applied; control groups consisted of untreated diabetic animals. These data helped to determine effect measurements and effect sizes of acupuncture’s effects on diabetes models. In cases where the data is presented entirely in graphical form, a concerted effort is made to obtain the necessary measurements. This requires contacting the study authors directly or using Get Data software to obtain the necessary values. In addition, the secondary outcomes are recorded in the form of mean ± standard deviation data. If the data is missing, we will contact the author of the article. If disputes arose, the final decision was reached through discussion and negotiation involving a third researcher.

### 2.4 Quality assessment

The methodological quality of the included literature was based on a 10-item checklist that had been adapted from the Collaborative Approach to Meta-Analysis and Review of Animal Data from Experimental Studies (CAMARADES) checklist (23). The checklist included the following criteria: publication in a peer-reviewed journal. Statements describing the control of temperature. Random allocation of subjects to treatment or control groups. Blinded construction of the model. Use of animals with hypertension or diabetes, if applicable. Blinded assessment of study outcomes. Utilization of anesthetic without marked intrinsic properties. Sample size calculation. Compliance with animal welfare regulations. Declaration of any potential conflicts of interest.

### 2.5 Statistics

In this study, we utilized STAT17.0 software for data analysis. For continuous variables, we calculated the standardized mean differences (SMD) along with their respective 95% confidence intervals (CI). Heterogeneity assessment was performed using the *I*² statistic. If *I*² was less than 50%, fixed-effects models were employed; for values exceeding 50%, random-effects models were applied. Sensitivity analysis was conducted to identify potential sources of heterogeneity. Subgroup analysis was carried out to explore heterogeneity sources, including model species, establishment of animal model, treatment duration, timing, acupuncture method, and organizational source. The publication bias of the selected study was scrutinized using Egger’s test.

## 3 Results

### 3.1 Research screening

**Figure 1:**
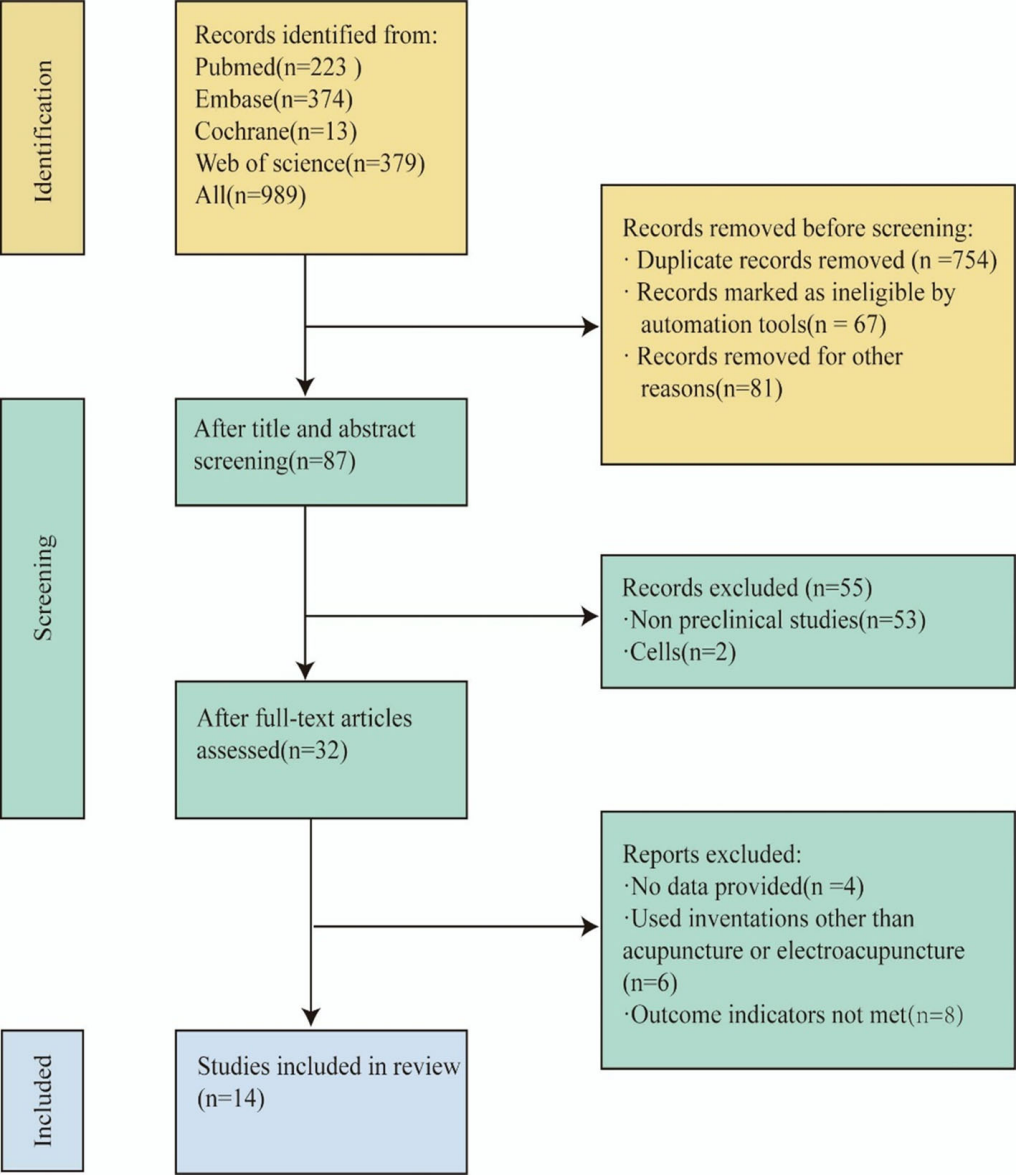
Literature screening flowchart.

Our research endeavor commenced with the implementation of a retrieval strategy. Two independent researchers (LLS, GZY) retrieved a total of 898 articles by searching in four databases (223 from PubMed, 374 from Embase, 379 from Web of Science, and 11 from Cochrane). After reading the titles, abstracts, and authors, and eliminating duplicates and irrelevant literature, 87 articles remained. By evaluating the titles and abstracts, we excluded 53 non-preclinical studies, and 2 studies specifically related to cells. Subsequently, a comprehensive examination of the full text was conducted for 32 articles. Among these, 4 articles met the inclusion criteria but did not provide data and were thus excluded. Additionally, 6 articles used interventions other than acupuncture and electroacupuncture, and 8 articles did not meet outcome indicators, leading to their exclusion as well. Ultimately, 14 studies were selected for a comprehensive analysis (15, 24–36) The process and results of literature selection are shown in **Figure 1**.

### 3.2 Research characteristic

**Figure 2:**
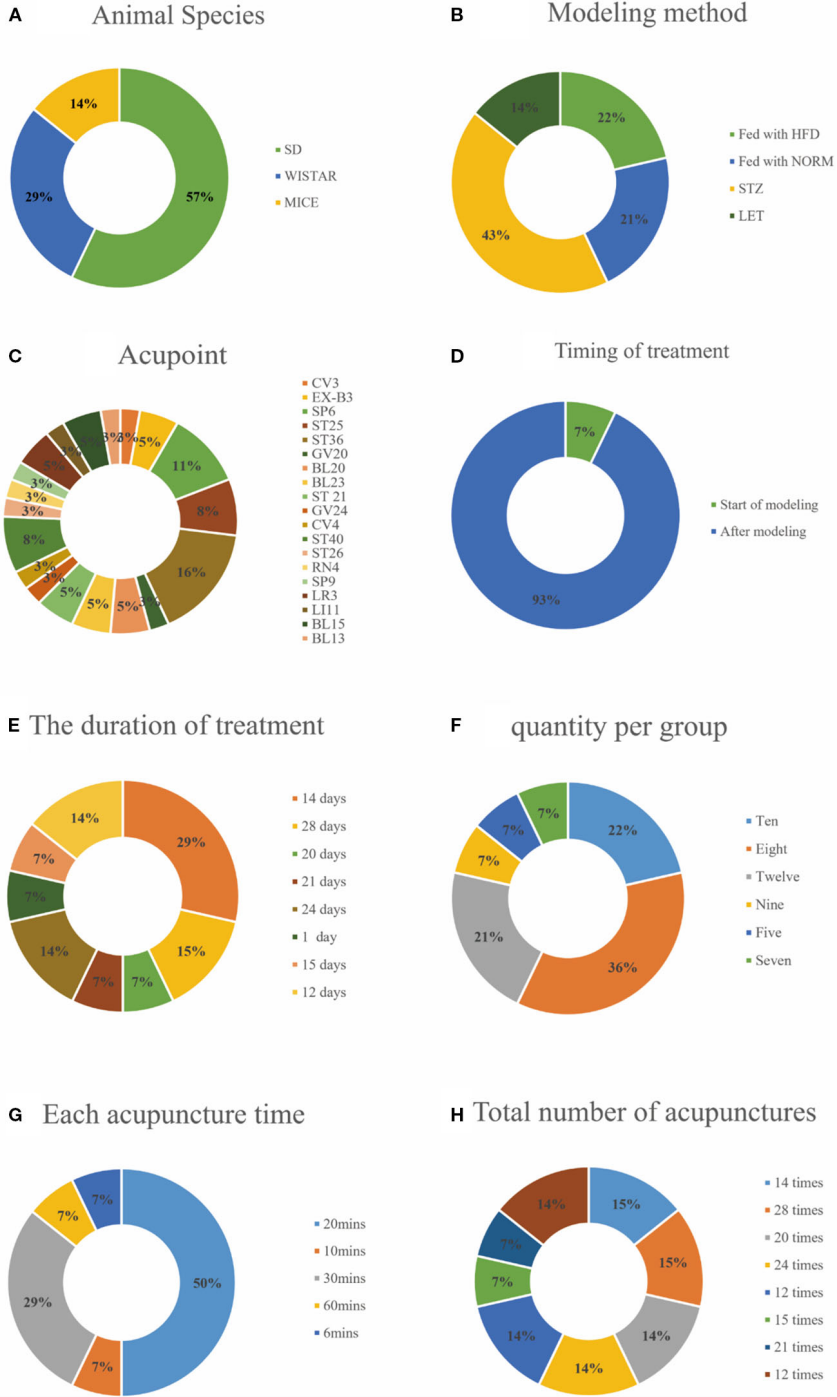
Included study characteristics. **(A)** Animal Species; **(B)** Modeling method; **(C)** Acupoint; **(D)** Timing of treatment; **(E)** The duration of treatment; **(F)** Quantity per group; **(G)** Each acupuncture time; **(H)** Total number of acupunctures.

The basic characteristics of the 14 selected studies are summarized in **Table 1**, A total of 274 animals were included in this study. Most of these animals were Sprague–Dawley rats, accounting for 57% of the sample, followed by Wistar rats at 29%, and Mus musculus at 14% (**Figure 2A**). Concerning the T2DM animal models used, all included studies used induced models of diabetes, including intraperitoneal streptozotocin (STZ) injection, high-fat diet feeding, or letrozole combined with high-fat diet. no spontaneous diabetes models (genetic models such as db/db mice) were included, with the intraperitoneal injection of STZ model being the most prevalent, featured in 43% of the studies, models fed with High-Fat Diet (HFD) made up 22%, models fed with NORM represent 21% and LET accounts for 14%. Each percentage reflects the proportion of studies using these specific methods (**Figure 2B**). Furthermore, there are significant differences in the selection of acupuncture points. ST36 acupuncture point was the most selected (16%), followed by SP6 (11%). CV4 and ST25 each account for 8%. Several other acupoints, including BL23, CV3, ST40, and EX-B3, each account for 5%. A variety of other acupoints, such as BL20, GV24, and LI11, each account for 3% (**Figure 2C**). Notably, In the context of timing and duration of treatment,93% of the treatments were administered after modeling, while only 7% of the treatments were administered at the start of modeling (**Figure 2D**). The duration of acupuncture treatment varied, ranging from 1 to 28 days, 24-days treatment durations were the most common, representing 29% of the studies (**Figure 2E**). The study showed that the number of animals in each group in the included literature varied, with groups of 8 making up the largest portion (36%), groups of 10,22%, and groups of 12,21%. Groups 9, 5 and 7 each accounted for 7% of the total (**Figure 2F**), It shows that 50% prefer a 20-minute treatment time, 29% choose a 30-minute treatment time, and 7% each prefer a 10-minute treatment time, a 60-minute treatment time, and a 6-minute treatment time (**Figure 2G**). For example, both the 28 times and 15 times acupuncture sessions account for 15%, while the 12 times, 21 times, and 20 times sessions represent 7%, 14%, and 14% (**Figure 2H**).

**Table 1:** Included in the list of basic characteristics of the literature.

Authors (Year)	Species	Modeling method	N/Per group	Method	Acupoint	Duration	Timing	Outcome
Jing Zhou (2023) (36)	SD Rats	LET(1.0 mg/kg)combine Fed with HFD	10	EA	CV3,CV4, ST40	14	After	1,2,3,4,5,6
Ting Pan (2023) (15)	Mus musculus	Fed with normal diet	8	EA	EX-B3, BL15	28	After	1,2,3,4,5,6
Zhixing Li (2018) (28)	SD Rats	Fed with HFD	8	EA	SP6, ST40	14	After	1,2,3
Shiya Huang (2023) (24)	SD Rats	LET(1.0 mg/kg)combine Fed HFD	8	EA	ST25, ST26	20	Start	1,2,3,4,5
Xiaomin Li (2021) (27)	SD Rats	Fed with HFD	10	EA	ST36, SP6, RN4, ST25	21	After	2,4,5,6
QIN Xihui (2023) (35)	SD Rats	Intraperitoneal injection of STZ(30 mg/kg)	10	EA	ST25, SP9, ST40, LR3, LI11	28	After	2,3,5
Huan-huan Tian (2018) (32)	SD Rats	Intraperitoneal injection of STZ(30 mg/kg)	12	EA	EX-B3, BL15,BL23	24	After	1,3,4,5,6
Mengyuan Li (2023) (26)	Mus musculus	Fed with normal diet	12	EA	GV20, GV24, BL13, BL20, LR3, SP6	24	After	1,2,3
Yu-Chen Lee (2011) (25)	Wistar Rats	Intraperitoneal injection of STZ(60 mg/kg)	8	EA	ST36	14	After	1
Hui-Ching Pai (2009) (29)	Wistar Rats	Intraperitoneal injection of STZ(60 mg/kg)	9	EA	ST36	1	After	1
Peng Yan (2017) (30)	SD Rats	Intraperitoneal injection of STZ(55 mg/kg)	12	EA	SP6, ST36, ST 21	15	After	1
Suhariningsih (2022) (31)	Mus musculus	Fed with normal diet	5	EA	BL20, BL23	12	After	1
Atsushi Tominaga (2021) (33)	Wistar Rats	Fed with HFD	7	EA	ST36	12	After	1,2
Chung-Yuh Tzeng (2015) (34)	Wistar Rats	Intraperitoneal injection of STZ(60 mg/kg)	8	EA	ST36	14	After	1

SD rats, Sprague-Dawley rats; STZ, streptozotocin; 1, blood glucose; 2, body weight; 3, food intake; 3, TC, total cholesterol; 4, TG, triglycerides;5, HDL-C, high-density lipoprotein cholesterol; 6, LDL-C, low-density cholesterol; EA, electroacupuncture; HFD, high-fat diet; LET, Letrozole.

### 3.3 Quality assessment

**Figure 3:**
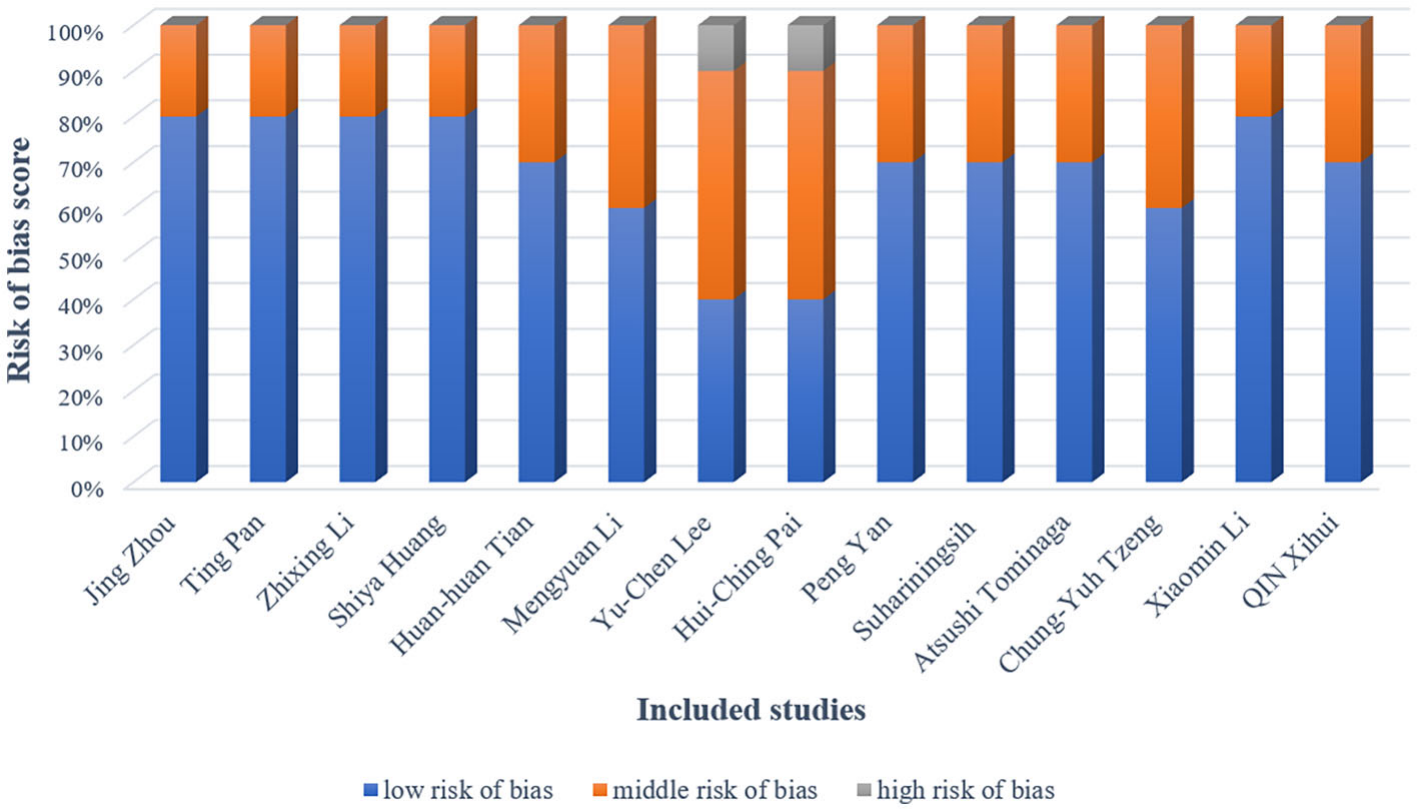
Risk of bias.

The methodological quality of included studies was rigorously evaluated using the CAMARADES checklist for bias assessment. **Figure 3** provides a detailed chart of the study quality assessment; In all studies, the basic characteristics of the animals were not significantly different. The overall quality of these studies was found to be moderate or higher. However, most of the studies did not describe the details of the trials in detail, especially regarding the randomization method and blinding. Blinding of the experimenter was not possible because the test groups were acupuncture, it is worth noting that two study did not detail the random allocation method. And one study did not describe all possible prognostic factors or animal characteristics.

### 3.4 Blood glucose

**Figure 4:**
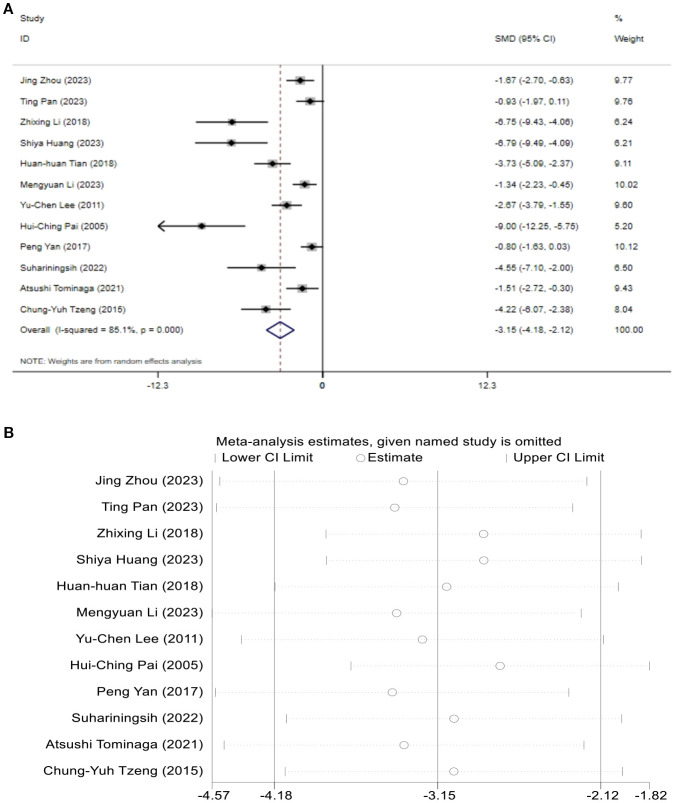
Forest plots **(A)** and sensitivity analysis **(B)** of blood glucose.

A total of 12 studies assessed the effect of BG. The results indicated that the levels of BG in the T2DM model group increased, while after acupuncture treatment, BG levels showed a significant reduction compared to the T2DM model group. [SMD = -3.15, 95% CI (-4.18, -2.12), *I^2^* = 85.1%] *(P* < 0.05) (**Figure 4A**). Sensitivity analysis showed that the combined effect estimates of all studies were within the confidence interval of the overall effect, indicating the reliability of the study results (**Figure 4B**).

### 3.5 Body weight

**Figure 5:**
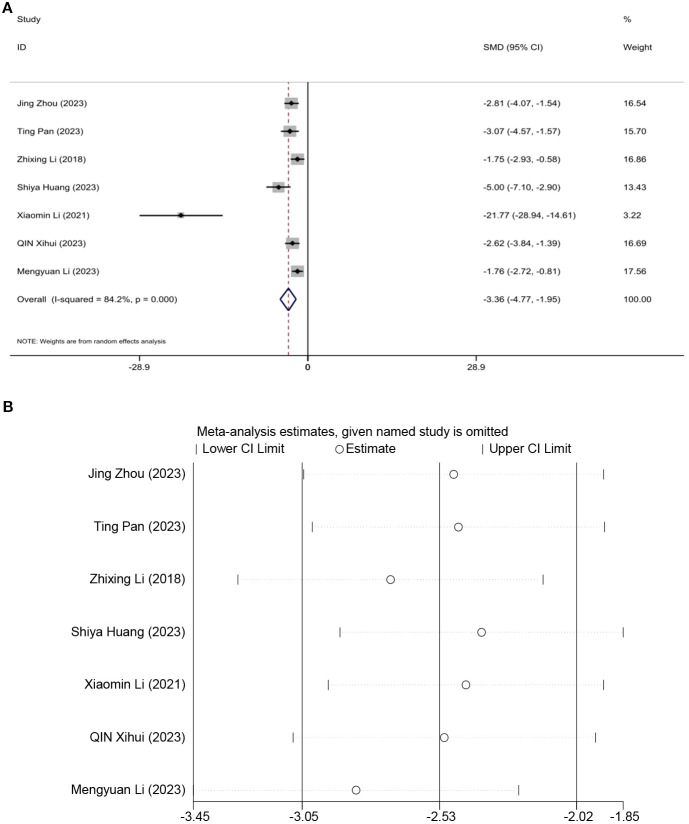
Forest plots **(A)** and sensitivity analysis **(B)** of body weight.

Seven studies assessed the effect of BW, demonstrating that acupuncture can reduce weight. A random effects model was used, and the meta-analysis showed that acupuncture significantly reduces BW in an animal model of T2DM. [SMD = -3.36, 95%CI (-4.77, -1.95), *I^2^* = 84.2%] (*P* < 0.05) (**Figure 5A**). Sensitivity analysis indicated that the combined effect estimates of all studies fell within the confidence interval of the overall effect, demonstrating the reliability of the study results (**Figure 5B**).

### 3.6 Triglyceide

**Figure 6:**
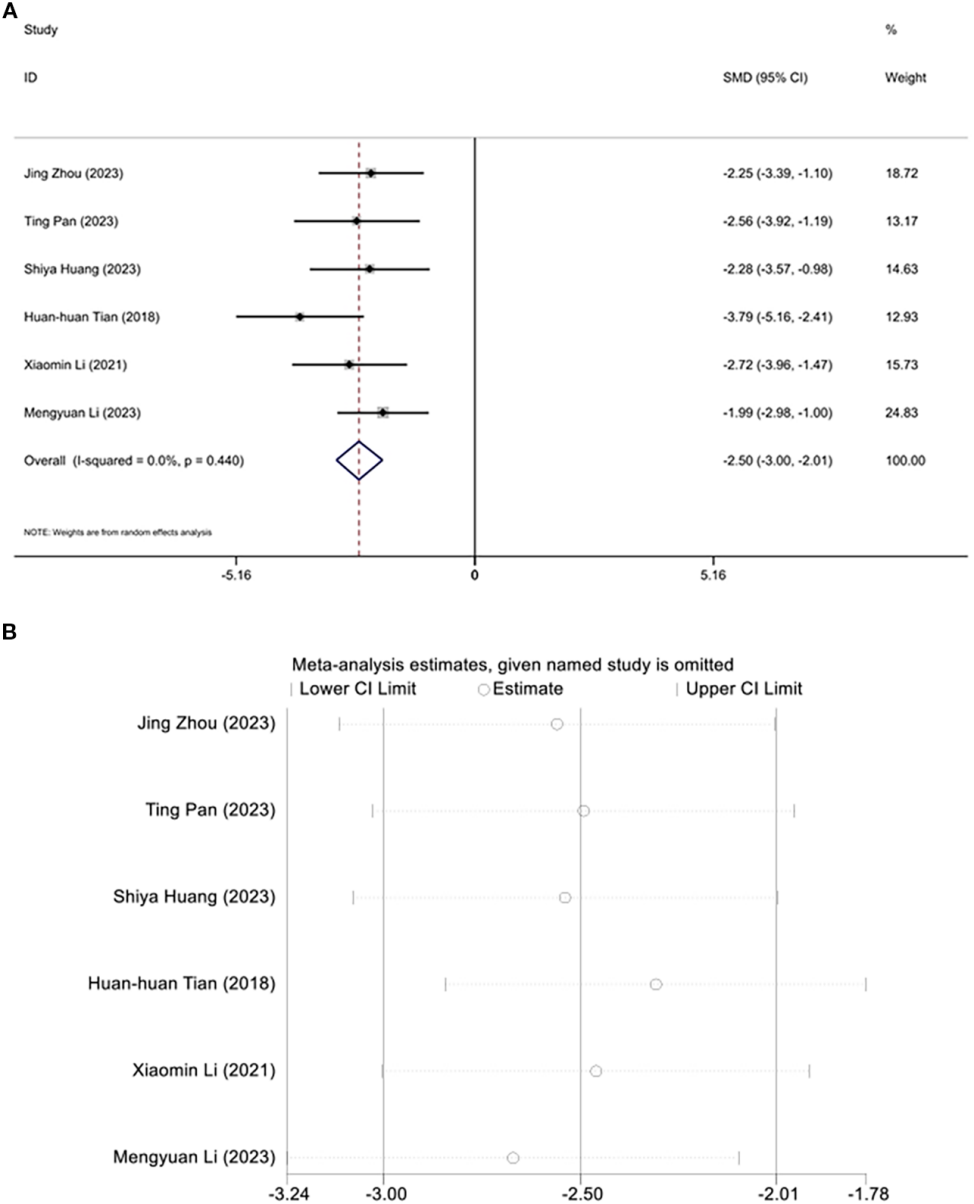
Forest plots **(A)** and sensitivity analysis **(B)** of Triglyceride.

Six studies investigated the regulation of triglyceride levels by acupuncture. Due to an *I*² value of less than 50, a fixed-effects model was applied for the analysis. Interestingly, following acupuncture treatment, a significant reduction in triglyceride levels was observed in the diabetic mouse model. Meta-analysis results indicated that acupuncture significantly reduced triglycerides in this animal model of T2DM. [SMD=-2.50, 95% CI (-3.00, -2.01), *I^2^* = 0.0%], (*P* < 0.05) (**Figure 6A**), and sensitivity analysis confirmed the robustness of the Triglyceride conclusion (**Figure 6B**).

### 3.7 Total cholesterol

**Figure 7:**
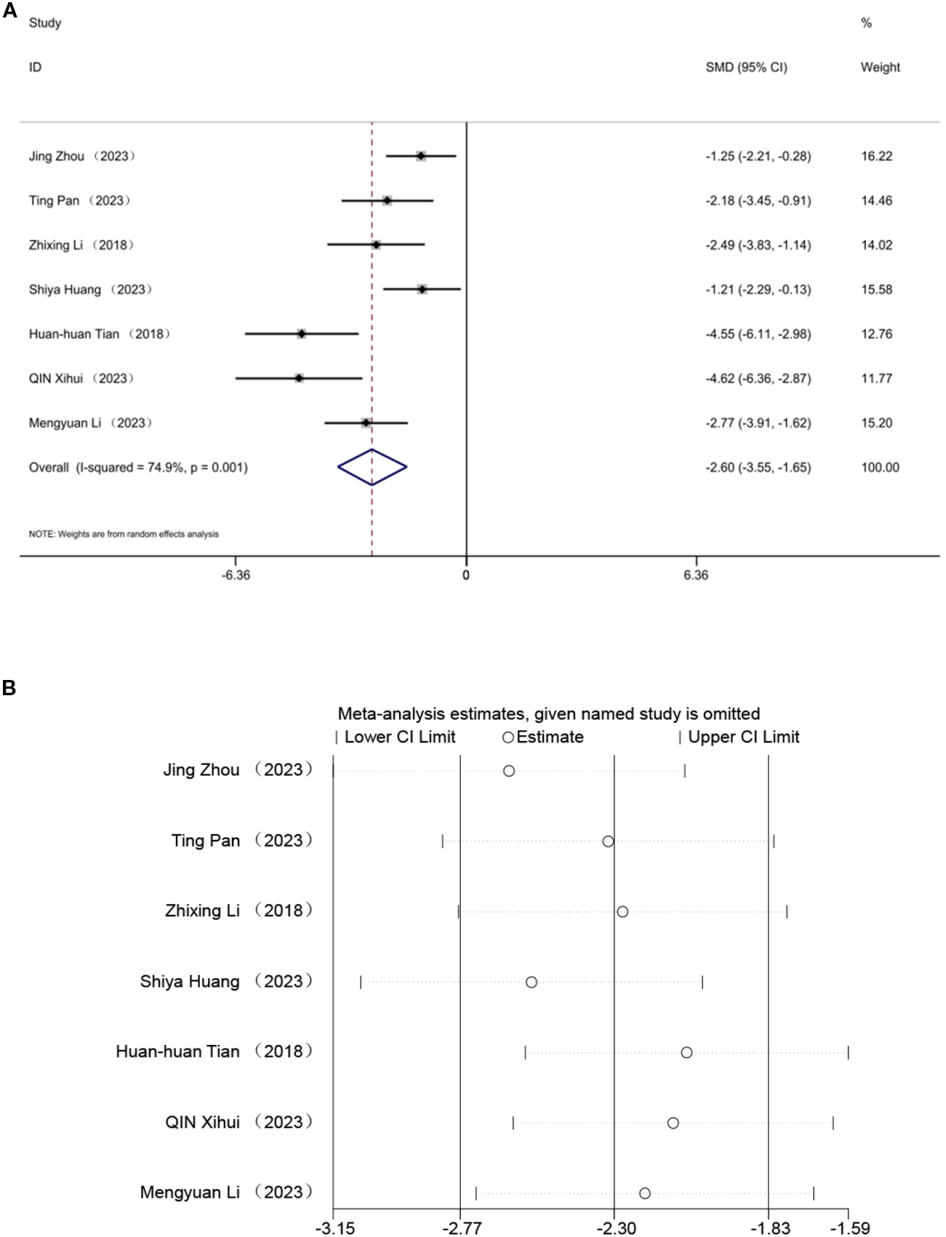
Forest plots **(A)** and sensitivity analysis **(B)** of total cholesterol.

Seven studies evaluated the effect of TC, indicating that acupuncture can lower TC levels. A random effects model was employed, and the meta-analysis revealed a significant reduction in TC with acupuncture in an animal model of T2DM. [SMD = -2.60, 95% CI (-3.55,-1.65), *I*² = 74.9%](*P* < 0.05) (**Figure 7A**). Sensitivity analysis further validated the strength of the LDL-C findings (**Figure 7B**).

### 3.8 High-density lipoprotein cholesterol

**Figure 8:**
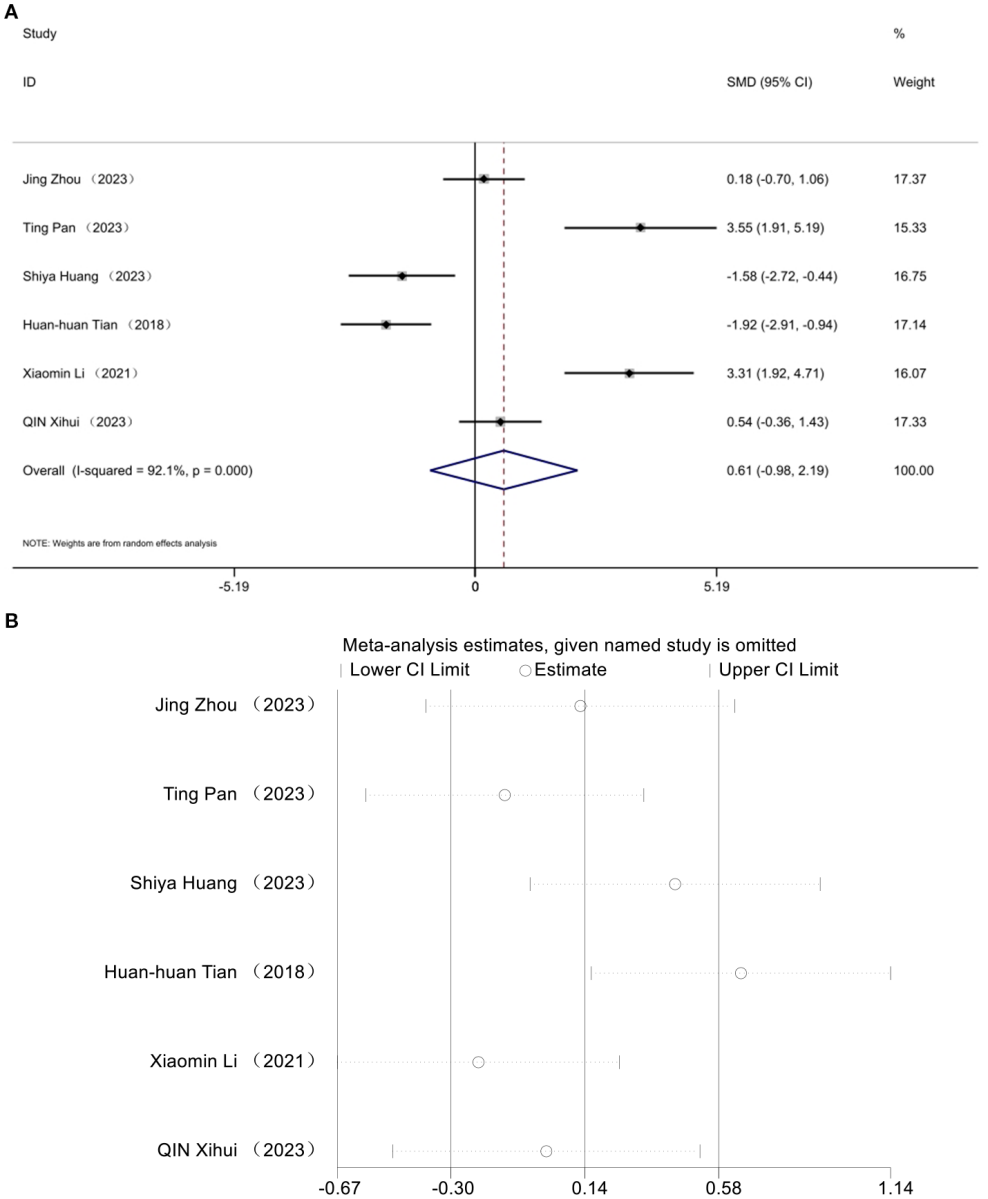
Forest plots **(A)** and sensitivity analysis **(B)** of high-density lipoprotein cholesterol.

The impact of HDL was documented in six studies. Analysis utilizing a random effects model revealed a notable decline in HDL levels within the diabetic mouse model [SMD = 0.61, 95% CI (-0.98, 2.19), *I*² = 92.1%] (*P*> 0.05) (**Figure 8A**). No statistically significant difference was observed in high-density lipoprotein cholesterol levels following acupuncture treatment in this T2DM animal model. Sensitivity analysis affirmed the robustness of the HDL findings (**Figure 8B**).

### 3.9 Low-density cholesterol

**Figure 9:**
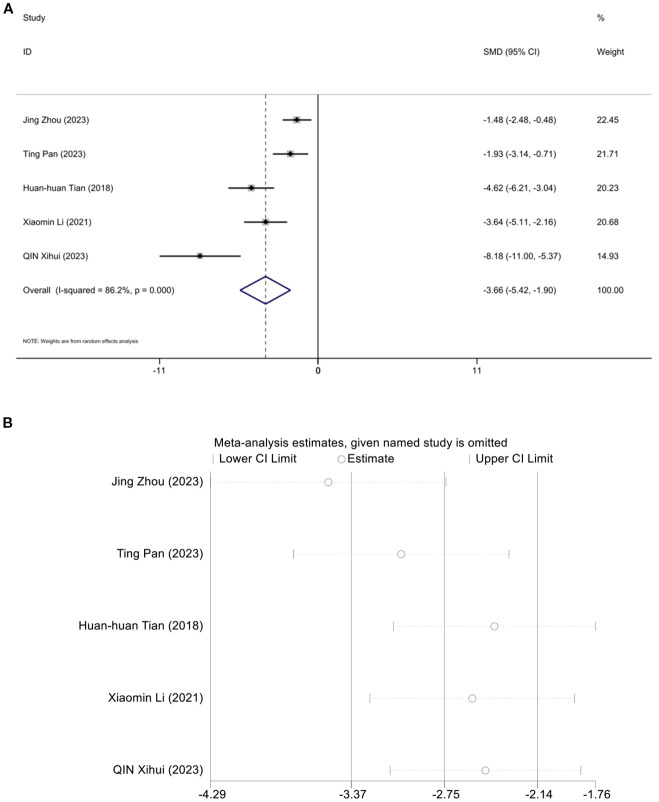
Forest plots **(A)** and sensitivity analysis **(B)** of low-density cholesterol.

Five studies assessed the effect of LDL-C, demonstrating that acupuncture can reduce LDL-C. A random effects model was used, and the meta-analysis showed that acupuncture significantly reduces LDL-C in an animal model of T2DM. [SMD = -3.36, 95%CI (-5.42,-1.95), *I^2^* = 86.2%] (*P* < 0.05) (**Figure 9A**). And sensitivity analysis confirmed the robustness of the LDL-C conclusion (**Figure 9B**).

### 3.10 Heterogeneity

**Figure 10:**
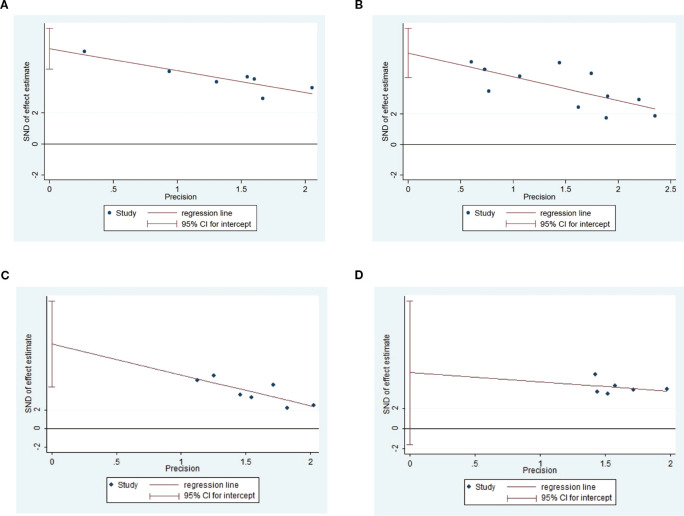
Egger’s test of Triglyceide **(A)**, total cholesterol **(B)**, body weight **(C)** and blood glucose **(D)**.

As shown in **Figure 10**, investigate the sources of heterogeneity, we selected four indicators: triglycerides (**Figure 10A**), total cholesterol (**Figure 10B**), body weight (**Figure 10C**), and blood glucose (**Figure 10D**), and conducted Egger’s test. The results of Egger’s test indicated the presence of publication bias in the included studies. Subsequently, we further analyzed potential sources of heterogeneity. Subsequently, we further analyzed the potential sources of heterogeneity. Animal species, acupuncture treatment duration, and timing of acupuncture intervention may be potential sources of heterogeneity. To further elucidate these sources, we performed a subgroup analysis on blood glucose, body weight, total cholesterol, and low-density lipoprotein levels.

### 3.11 subgroup analysis

#### 3.11.1 Blood glucose

**Figure 11:**
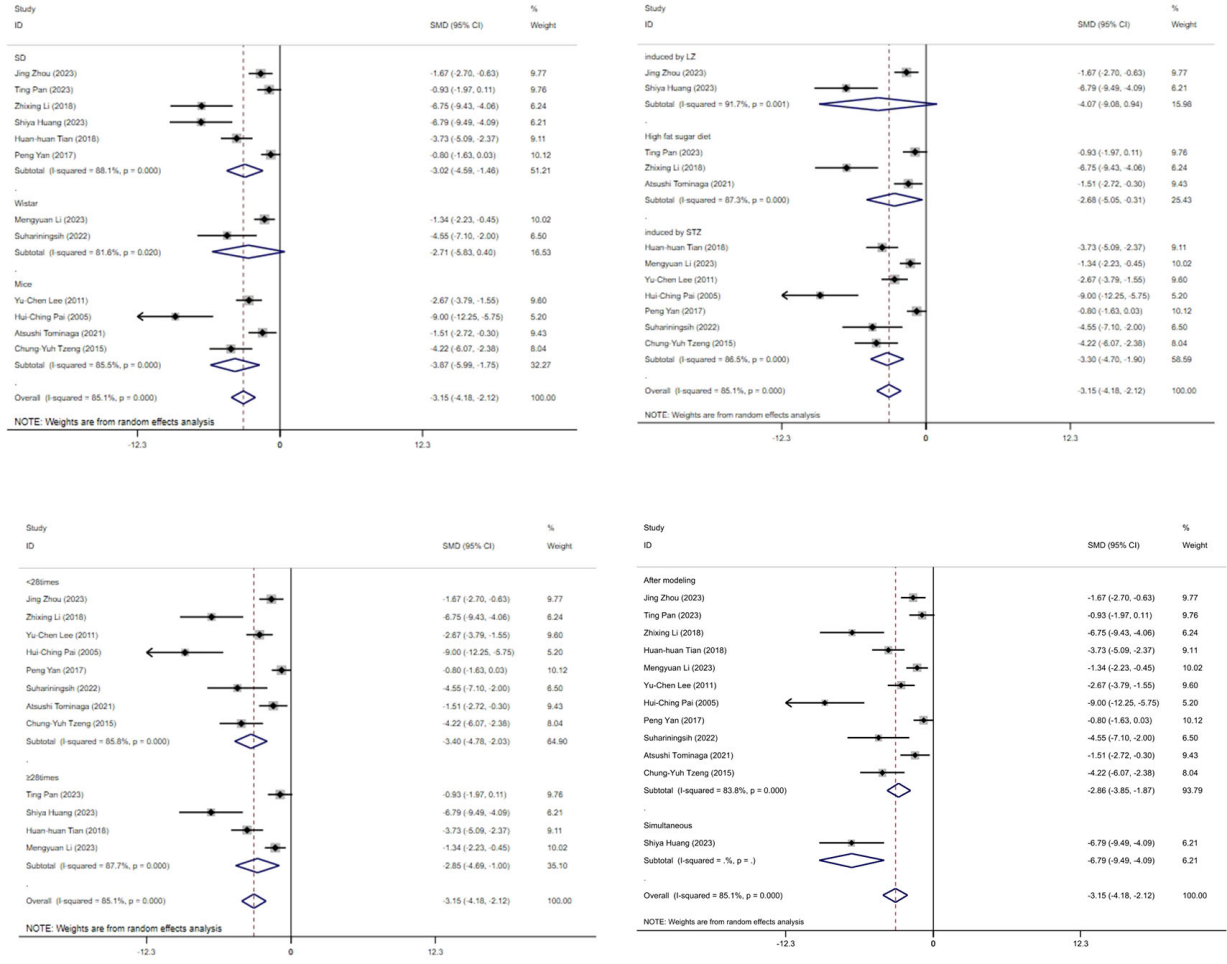
Subgroup analysis of blood glucose. **(A)** Model species; **(B)** Establishment of animal model; **(C)** The duration of treatment; **(D)** Timing of treatment.

**Figure 11** displays subgroup analyses of blood glucose levels stratified by model species (A), animal model establishment (B), treatment duration (C), and treatment timing (D), Subgroup meta-analysis of the model species (SD rats, Wistar rats, and mice) in the test group showed that the SD rat subgroup. [SMD = -3.02,95% CI (-4.59, -1.46); *I*² = 88.1% *(P* < 0.001)]; for the Wistar rat subgroup.[SMD = -2.71,95% CI (-5.83, 0.40); *I*² = 81.6% *(P* < 0.05)]; and for the mice subgroup.[SMD= -3.87 [95% CI (-5.99, - 1.75); *I*² = 85.5% *(P* < 0.001)] (**Figure 11A**). Subgroup meta- analysis of induction methods (LZ, high - fat sugar diet, STZ) showed that for LZ-induced subgroup. [SMD = -4.07, 95% CI (-9.08, 0.94), *I*² = 91.7% *(P* < 0.01)]; for high - fat sugar diet subgroup, [SMD = -2.68, 95% CI (-5.05, -0.31), *I*² = 87.3% *(P* < 0.001)]; for STZ-induced subgroup. [SMD = -3.30, 95% CI (-4.70, -1.90), *I*² = 86.5% *(P* < 0.001)] (**Figure 11B**). Subgroup meta-analysis of gestational age groups (<28times and ≥28times) showed that for the <28times subgroup. [SMD = -3.40, 95% CI (-4.78, -2.03); *I*² = 85.8% (*P*< 0.001)]; and for the ≥28times subgroup. [SMD = -2.85, 95% CI (-4.69, -1.00); *I*² = 87.7% (*P*< 0.001)] (**Figure 11C**). Subgroup meta-analysis of studies by modeling method showed that for the “After modeling” subgroup, [SMD = -2.86, 95% CI (-3.85, -1.87); *I*² = 83.8% (*P* < 0.001)]; and for the “Simultaneous” subgroup, [SMD = -6.79, 95% CI (-9.49, -4.09) (**Figure 11D**).

#### 3.11.2 Body weight

**Figure 12:**
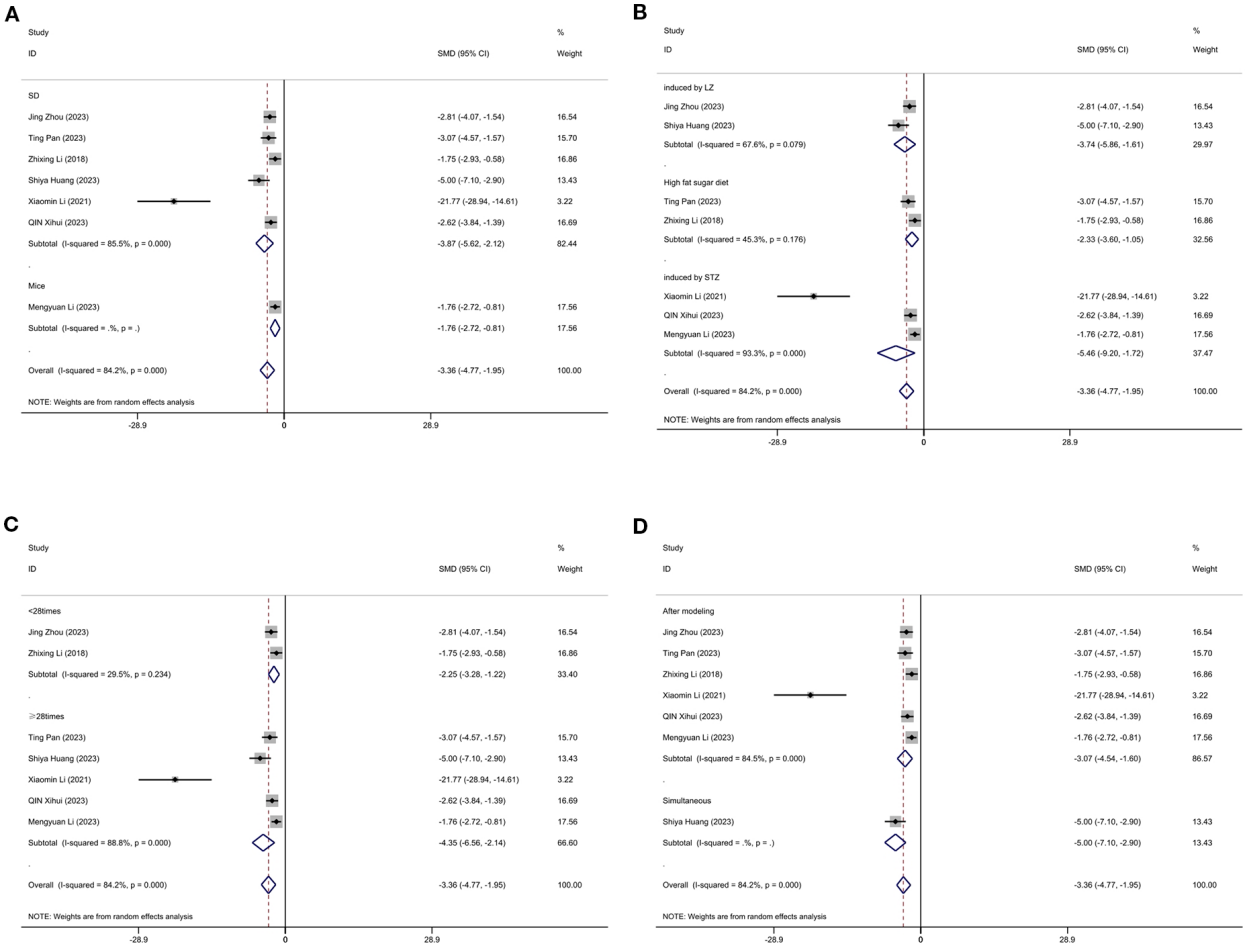
Subgroup analysis of body weight. **(A)** Model species; **(B)** Establishment of animal model; **(C)** The duration of treatment; **(D)** Timing of treatment.

**Figure 12** displays subgroup analyses of body weight stratified by model species (A), establishment of animal model (B), the duration of treatment (C)and timing of treatment (D). Subgroup meta-analysis of the model species (SD rats, mice) in the test group showed that for the SD rat subgroup, [SMD = -3.87, 95% CI (-5.62, -2.12); *I*² = 85.5% (*P* < 0.001)]; and for the mice subgroup, [SMD = -1.76, 95% CI (-2.72, -0.81)] (**Figure 12A**). Subgroup meta-analysis of induction methods (LZ, high-fat sugar diet, STZ) showed that for the LZ-induced subgroup, [SMD = -3.74, 95% CI (-5.86, -1.61); *I*² = 67.6% (*P* = 0.079)]; for the high-fat sugar diet subgroup, [SMD = -2.23, 95% CI (-3.60, -1.05); *I*² = 45.3% (*P* = 0.176)]; and for the STZ -induced subgroup, [SMD = -5.46, 95% CI (-9.20, -1.72); *I*² = 93.3% (*P* < 0.001)] (**Figure 12B**). Subgroup meta-analysis of time point groups (<28times, ≥28times) showed that for the <28 times subgroup, [SMD = -3.40, 95% CI (-4.78, -2.20); *I*² = 85.8% (*P* < 0.001)]; and for the ≥28times subgroup, [SMD = -4.35, 95% CI (-6.56, -1.14); *I*² = 88.8% (*P* < 0.001)] (**Figure 12C**). Subgroup meta - analysis of studies by modeling method showed that for the “After modeling” subgroup, [SMD = -3.07, 95% CI (-4.54, -1.60); *I*² = 84.5% (*P* < 0.001)]; and for the “Simultaneous” subgroup, [SMD = -5.00, 95% CI (-7.10, -2.90) (**Figure 12D**).

#### 3.11.3 Total cholesterol

**Figure 13:**
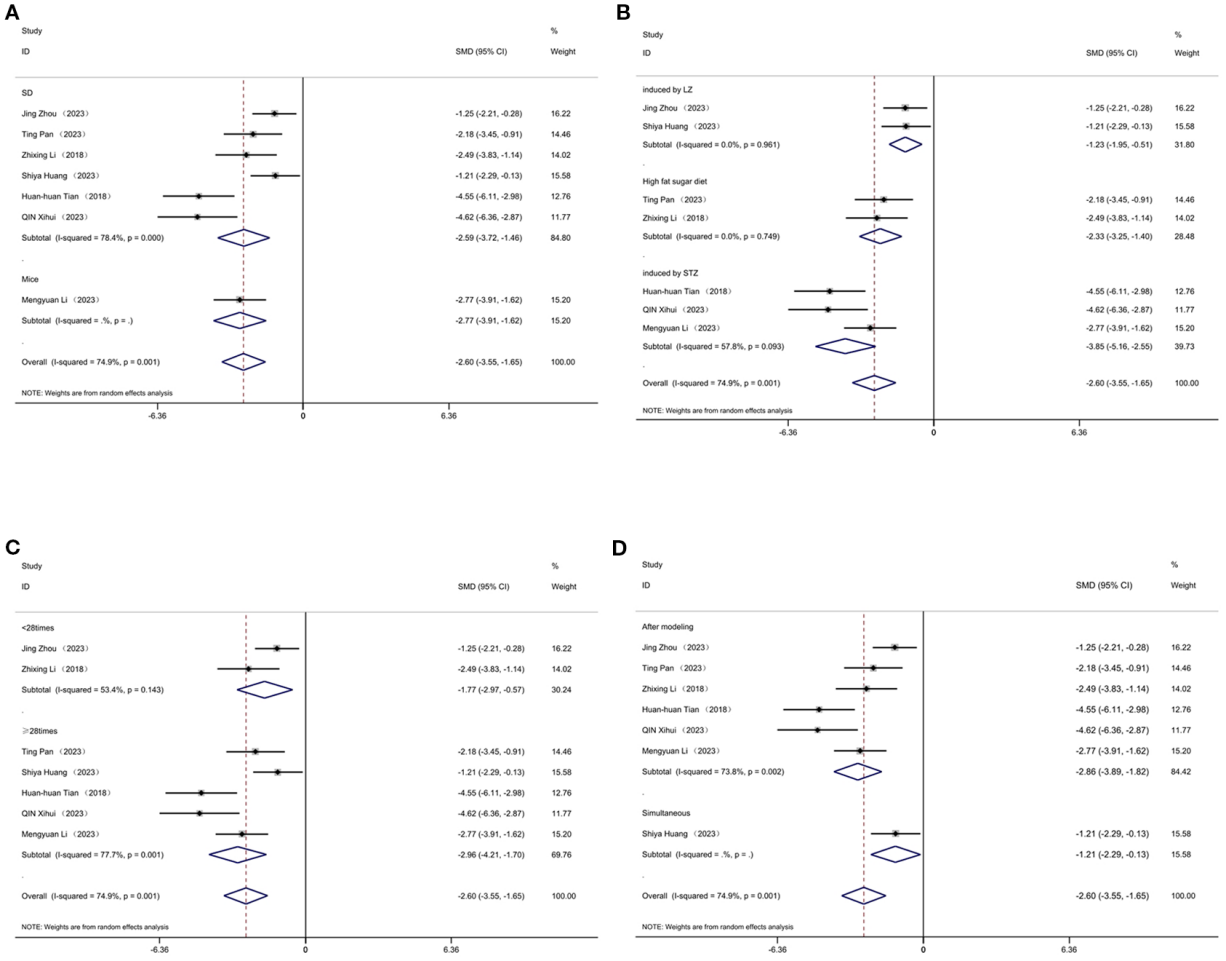
Subgroup analysis of cholesterol. **(A)** Model species; **(B)** Establishment of animal model; **(C)** The duration of treatment; **(D)** Timing of treatment.

**Figure 13** displays subgroup analyses of total cholesterol stratified by model species (A), establishment of animal model (B), the duration of treatment (C) and timing of treatment (D). Subgroup meta-analysis of the model species (SD, mice) in the test group showed that for the SD subgroup, [SMD = -2.59, 95% CI (-3.72, -1.46); *I*² = 78.4% (*P* < 0.001)]; and for the mice subgroup, [SMD = -2.77, 95% CI (-3.91, -1.62)] (**Figure 13A**). Subgroup meta - analysis of establishment methods (induced by LZ, high fat sugar diet, induced by STZ) showed that for the LZ-induced subgroup, [SMD = -1.23, 95% CI (-1.95, -0.51); *I*² = 0% (*P* = 0.961)]; for the high fat sugar diet subgroup, [SMD = -2.33, 95% CI (-3.25, -1.40); *I*² = 0% (*P* = 0.749)]; and for the STZ - induced subgroup, [SMD = -3.85, 95% CI (-5.16, -2.55); *I*² = 57.8% (*P* = 0.093)] (**Figure 13B**). Subgroup meta - analysis of treatment duration groups (<28 times, ≥28 times) showed that for the <28 times subgroup, [SMD = -1.77, 95% CI (-2.97, -0.57); *I*² = 53.4% (*P* = 0.143)]; and for the ≥28 times subgroup, [SMD = -2.96, 95% CI (-4.21, -1.70); *I*² = 77.7% (*P* < 0.001)] (**Figure 13C**). Subgroup meta-analysis of studies by treatment timing showed that for the “After modeling” subgroup, [SMD = -2.86, 95% CI (-3.89, -1.82); *I*² = 73.8% (*P* < 0.01)]; and for the “Simultaneous” subgroup, [SMD = -1.21, 95% CI (-2.29, -0.13)] (**Figure 13D**).

#### 3.11.4 Low-density cholesterol

**Figure 14:**
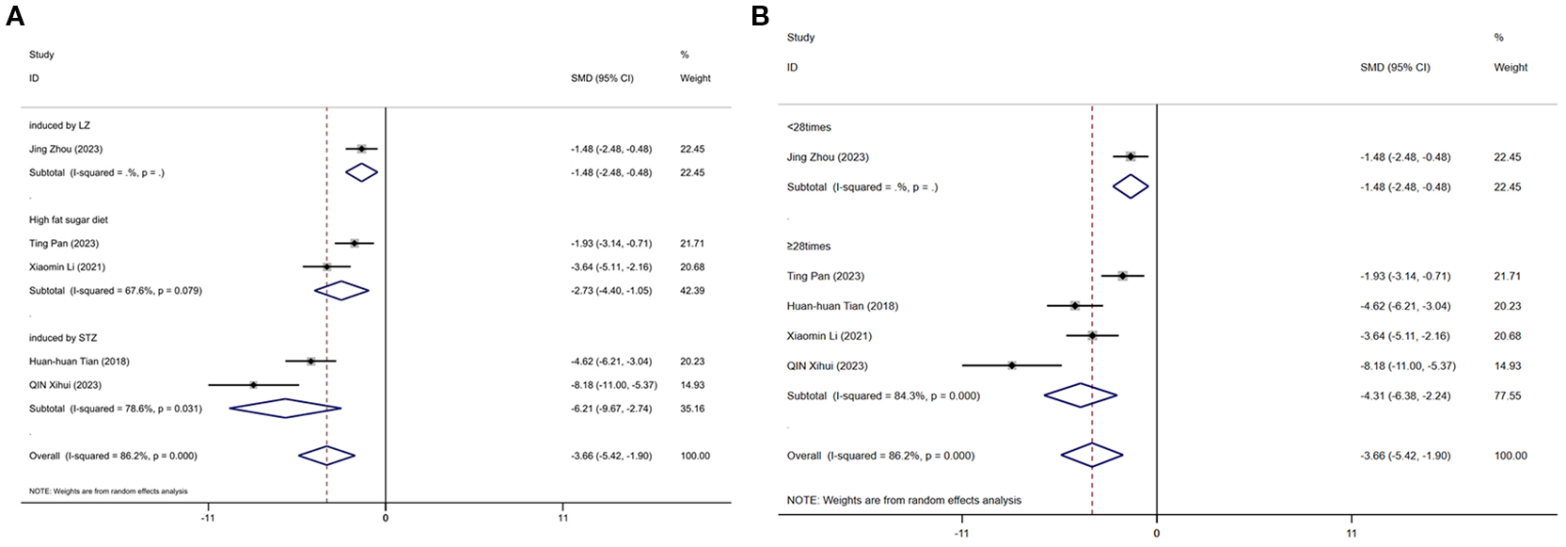
Subgroup analysis of blood Low-density cholesterol. **(A)** Establishment of animal model; **(B)** The duration of treatment.

**Figure 14** displays subgroup analyses of blood LDL stratified by establishment of animal model (A) and the duration of treatment (B). Subgroup meta-analysis of the establishment of animal model (induced by LZ, high fat diet, induced by STZ, high fat sugar diet) showed that for the induced by LZ subgroup, [SMD = -1.48, 95% CI (-2.48, -0.48)]; for the high fat diet subgroup, [SMD = -2.73, 95% CI (-4.40, -1.05); *I*² = 67.6% (*P* = 0.079)]; and for the induced by STZ subgroup, [SMD = -6.21, 95% CI (-9.67, -2.74); *I*² =78.6% (*P* = 0.031)] (**Figure 14A**). Subgroup meta-analysis of the duration of treatment groups (<28times, ≥28times) showed that for the <28times subgroup, [SMD = -1.48, 95% CI (-2.48, -0.48)]; and for the ≥28times subgroup, [SMD = -4.31, 95% CI (-6.38, -2.24); *I*² = 84.3% (*P* < 0.001)] (**Figure 14B**).

## 4 Discussion

### 4.1 Principle findings

The objective of this research was to summarize and evaluate the use of acupuncture to improve blood glucose and lipid levels in the animal model of T2DM. In this study, we systematically gathered information from 14 acupuncture studies animal models of T2DM to determine, whether acupuncture can improve blood glucose and lipid levels, we found that acupuncture was closely associated with decreased BW, TC, TG and LDL, that acupuncture could significantly improve the level of blood glucose in animal with T2DM, which confirms previous reports (37). Diabetes is a group of metabolic disorders characterized by chronically elevated blood glucose levels. It disrupts glucolipid metabolism, resulting in dyslipidemia, which elevates the risk of cardiovascular disease. Glucolipid metabolism disorders cause serum levels of TC, TG and LDL-C to rise (38, 39). The meta-analysis revealed that compared to the model group, acupuncture significantly reduced blood glucose levels in diabetic animals. Both the experimental and model groups exhibited hyperglycemia prior to acupuncture treatment, but the hyperglycemic condition in the experimental group improved following acupuncture intervention; therefore, the meta-analysis showed that acupuncture intervention in T2DM animal models could effectively reduce the serum levels of TC, TG, and LDL-C and improve the lipid metabolism of T2DM animals to some extent. However, there was high heterogeneity in the analysis results, Subgroup analysis showed that intervention time, modeling methods, animal species, and duration were sources of heterogeneity.

Subgroup analyses yielded valuable insights. For body weight and cholesterol, subgroups with high-fat diet (HFD) models or combined HFD and STZ models generally exhibited larger effect sizes compared to those with letrozole-induced models, indicating that the underlying pathophysiology of the animal model can significantly influence the responsiveness to acupuncture treatment. Likewise, treatment durations shorter than 28 days seemed to result in more consistent improvements in body weight, which may be attributed to the acute-phase responsiveness of metabolic parameters in animal models. These observed trends are in line with previous research that found electroacupuncture had greater insulin-sensitizing effects in diet-induced insulin resistance models than in chemically induced β-cell injury models (40), and with Wang et al. (41) reporting that the modulation of gut microbiota by acupuncture was more prominent in HFD-fed mice, supporting our finding of model-dependent variability. Species selection did not emerge as a major factor determining the effect size. This is consistent with the understanding that the metabolic and neuroendocrine pathways targeted by acupuncture are conserved across rodent models (42). However, species-specific differences in lipid metabolism need to be taken into account in translational research.

Our findings emphasize the significance of carefully selecting appropriate animal models and optimizing treatment duration in preclinical acupuncture studies. These two parameters may also have implications for the design of clinical protocols, as the patient’s metabolic phenotype and the treatment course could potentially influence therapeutic outcomes.

### 4.2 Mechanistic insights into acupuncture for T2DM

#### 4.2.1 Neural mechanisms

The autonomic nervous system regulates pancreatic islet function through sympathetic and parasympathetic innervation. Parasympathetic activity promotes insulin secretion, whereas sympathetic input generally exerts inhibitory effects. Acupuncture has been shown to modulate the pancreatic intrinsic nervous system (PINS) and associated neuropeptides, such as Substance P and CGRP, thereby improving β-cell function (43). Electroacupuncture at ST25 has also been reported to enhance TRPV1 pathway activity, leading to improved glucose metabolism (13). Moreover, transcutaneous auricular vagus nerve stimulation reduces food intake and weight gain in high-fat diet mice via an orexin-dependent pathway (K. 44), suggesting that acupuncture exerts neuromodulatory effects on energy metabolism. Collectively, acupuncture may regulate β-cell function through vagus nerve activity, PINS, and neuropeptide signaling, while auricular vagus pathways may modulate appetite and body weight, highlighting the importance of neural mechanisms in acupuncture-mediated intervention for diabetes.

#### 4.2.2 Endocrine mechanisms

Acupuncture improves insulin sensitivity and glucose uptake by modulating the PI3K/AKT/GLUT4 signaling pathway (45, 46). This effect involves the upregulation of IRS-1, PI3K, AKT, and GLUT4 expression, while also enhancing mitochondrial function and energy metabolism through the AMPK/SIRT1/PGC-1α axis (47). In addition, acupuncture regulates lipid metabolism via the AMPK/ACC pathway, producing significant metabolic benefits in diabetes complicated with non-alcoholic fatty liver disease (NAFLD) (48). Furthermore, acupuncture influences the BDNF pathway, thereby ameliorating comorbid diabetes and depression (44), suggesting a dual regulatory effect on the metabolic–neuroendocrine axis. Collectively, acupuncture acts through PI3K/AKT/GLUT4 and AMPK-related pathways to enhance insulin sensitivity and energy metabolism, while also modulating BDNF signaling to improve metabolic and neuropsychiatric comorbidities.

#### 4.2.3 Immune mechanisms

Inflammatory cytokines and immune dysregulation play pivotal roles in the pathogenesis of diabetes. SIRT1 reduces the expression of inflammatory factors such as TNF-α and IL-6 by inhibiting NF-κB signaling, thereby ameliorating insulin resistance (49). Acupuncture may exert anti-inflammatory effects via this mechanism (50). Additionally, acupuncture can regulate immune–metabolic crosstalk through miRNA modulation. For instance, acupuncture treatment protects patients with diabetes and polycystic ovary syndrome (PCOS) via the miR-32-3p/PLA2G4A pathway (51). Furthermore, acupuncture mitigates inflammatory responses and immune-related damage in diabetic complications, such as diabetic nephropathy and cognitive impairment (52). Thus, acupuncture exerts beneficial effects on diabetes and its complications by modulating inflammation and immunity through SIRT1/NF-κB and miRNA-related pathways.

#### 4.2.4 Gut microbiota-related mechanisms

The gut microbiota is closely linked to metabolic diseases. EA improves insulin resistance by reshaping gut microbial composition, such as increasing the abundance of Akkermansia (15). In addition, acupuncture regulates gut–brain axis function via the PI3K/Akt pathway, influencing ghrelin and peptide YY expression, thereby affecting feeding behavior and energy metabolism (46). Acupuncture also enhances gut barrier integrity by upregulating tight junction proteins, reducing inflammation, and mitigating metabolic dysregulation (53). These findings suggest that acupuncture exerts antidiabetic effects by modulating gut microbiota composition, improving intestinal barrier function, and regulating gut–brain axis signaling, ultimately restoring glucose and lipid homeostasis.

#### 4.2.5 Autophagy-related mechanisms

Dysregulated autophagy is strongly associated with diabetes onset and progression. Acupuncture restores hepatic autophagy through the AMPK/mTOR pathway, thereby ameliorating NAFLD associated with diabetes (45). In the nervous system, acupuncture enhances mitochondrial autophagy, improving cognitive dysfunction in diabetes. Moreover, acupuncture modulates endoplasmic reticulum stress and autophagy processes to attenuate cognitive impairment in diabetic models (54). Collectively, acupuncture activates AMPK/mTOR and mitochondrial autophagy pathways to alleviate diabetes-related hepatic and neurological damage, underscoring the importance of autophagy in acupuncture therapy for diabetes.

#### 4.2.6 Advances and limitations

Research on acupuncture for diabetes has gradually expanded from clinical efficacy observation to mechanistic studies at the molecular, neuroendocrine, immunological, gut microbiota, and autophagy levels. Evidence indicates that acupuncture exerts multi-target and multi-pathway regulatory effects: modulating central and autonomic neural circuits, enhancing insulin signaling, regulating immune–inflammatory responses, reshaping gut microbiota, and restoring autophagic balance. Nevertheless, current studies face limitations. Most findings are derived from animal models or small-scale clinical trials, lacking large-scale, multicenter randomized controlled trials to confirm reproducibility and clinical value. Moreover, although classical pathways such as PI3K/Akt, AMPK/mTOR, and NF-κB have been implicated, discrepancies remain due to differences in study design and acupoint selection (46, 48, 51). Individual variations in gut microbiota and immune regulation also suggest that acupuncture efficacy may have personalized characteristics, but precision-medicine–oriented research is still lacking. Furthermore, standardized treatment protocols, dose–response relationships, and long-term follow-up studies remain underdeveloped.

In conclusion, acupuncture demonstrates a unique advantage in integrative, multi-level regulation for T2DM management. Future research should focus on conducting large-scale RCTs, applying multi-omics approaches (transcriptomics, metabolomics, microbiomics), investigating individual variability to advance precision acupuncture strategies, and integrating neuroimaging and neuromodulation techniques to further elucidate central mechanisms of acupuncture.

### 4.3 Implications for further research

T2DM is a prevalent metabolic disorder characterized by endocrine dysregulation arising from multifactorial pathogenesis (10). Within this framework, closely intertwined insulin resistance and dyslipidemia constitute key drivers of disease progression (55). Our meta-analysis demonstrates acupuncture’s efficacy in modulating glycemic and lipidemic profiles in diabetic rodent models. These findings align with recent mechanistic evidence: Luo et al.’s (56) systematic review identified insulin signaling potentiation and AMPK-mediated mitochondrial biogenesis as core pathways underlying acupuncture’s antidiabetic effects. While our study primarily evaluates metabolic outcomes, this mechanistic coherence enhances the biological plausibility of our results (56). Nevertheless, several limitations in current experimental designs warrant acknowledgment. Firstly, most studies focus on Type 2 Diabetes Mellitus, with relatively few investigations addressing Type 1 Diabetes Mellitus. Additionally, while induced models are frequently utilized, spontaneous models are less common. The complexity of the pathogenesis of human diabetes poses challenges in replicating its symptoms and underlying mechanisms, as it is influenced by various factors. Therefore, there is a need to improve relevant animal models and conduct clinical research to explore the efficacy of acupuncture in treating diabetes. Secondly, on the clinical front, although acupuncture shows potential for managing diabetes, the underlying molecular mechanisms remain unclear. Most existing research revolves around acupuncture treatment for T2DM; however, there is a lack of direct evidence supporting the effectiveness of acupuncture in improving blood glucose levels in patients with T1DM. While many studies indicate beneficial effects of acupuncture for diabetic patients, the majority are non-randomized controlled trials with small sample sizes and limited scope. There is a notable absence of large-scale, multi-center randomized controlled trials demonstrating the efficacy and safety of acupuncture in diabetes management.

### 4.4 Strengths and limitations of the research

To our knowledge, our study presents the first systematic review and meta-analysis of the efficacy of acupuncture in treating animal models of DM, which provides scientific evidence that acupuncture can improve blood glucose and lipid levels in these models. Although there was heterogeneity among the results of the analyzed studies, exploratory meta-regression suggested that smaller sample sizes might contribute to variability in effect estimates, although statistical significance was not reached. This aligns with the general observation that underpowered studies may yield unstable results, underscoring the need for larger preclinical experiments. The study is subject to the following limitations: Firstly, the search was restricted to only English-language databases, potentially limiting the comprehensiveness of the systematic evaluation due to the restricted pool of studies. Secondly, the exclusion of non-English language studies may hinder a comprehensive understanding of acupuncture’s efficacy in T2DM treatment, potentially impacting the accuracy of the analysis results. Thirdly, the lack of description regarding blinding in animal experiments and the exclusive focus on murine species in the included literature may introduce a risk of false positive results. While our study has its scope limitations, the analysis of blood glucose and lipid metabolism allows for the first assessment of acupuncture’s efficacy in the animal model of T2DM, which carries meaningful implications. Sex information was missing in most included studies; where reported, male animals were predominantly used, potentially limiting generalizability. Future studies should follow the NIH ‘Sex as a Biological Variable’ policy to ensure balanced representation and transparent reporting. As a traditional medical treatment, corresponding measures of variability acupuncture has not yet established and internationally accepted standardized treatment protocol, which hinders its widespread application in clinical settings. This issue must be addressed in future work. It is undeniable that our research has certain limitations. There exists heterogeneity in the design of animal studies and clinical trials; although subgroup analyses were conducted, the sources of this heterogeneity remain unclear. Furthermore, not all included studies are of high quality, which may affect the assessment of acupuncture’s efficacy. The implementation of blinding in acupuncture studies also presents challenges. While factors such as sex and age play significant roles in the development of diabetes, very few experimental designs take these variables into account. Therefore, it is recommended that future experimental research standardize protocols, considering potential influencing factors such as sex, age, and disease stage. This will facilitate the exploration of different acupuncture treatment methods for diabetes and provide a solid preliminary foundation for large-scale, multi-center high-quality randomized controlled trials.

## 5 Conclusion

A systematic review and meta-analysis have revealed that acupuncture confers notable protective benefits for animals with T2DM. When compared to control groups, acupuncture significantly improved blood glucose, body weight, total cholesterol, triglycerides, and low-density lipoprotein cholesterol levels in animals with diabetes mellitus. Nonetheless, this study faces several limitations stemming from the inadequate number of included studies, methodological shortcomings, and insufficient sample sizes. To fully elucidate acupuncture’s effects in T2DM animals, further research involving larger samples and higher-quality methodologies is imperative. We look forward to future investigations exploring the therapeutic potential of acupuncture and trust that this review will serve as a valuable reference in advancing this area of study.

## Data Availability

The original contributions presented in the study are included in the article/Supplementary Material, further inquiries can be directed to the corresponding author/s.
